# *Aspergillus* ball crawling out of pulmonary bullae

**DOI:** 10.1111/1759-7714.14752

**Published:** 2022-12-04

**Authors:** Yuhui Liu, Hongtu Yuan, Yong Huang

**Affiliations:** ^1^ Department of Radiology, Shandong Cancer Hospital and Institute Shandong First Medical University and Shandong Academy of Medical Sciences Jinan People's Republic of China; ^2^ Department of Pathology, Shandong Cancer Hospital and Institute Shandong First Medical University and Shandong Academy of Medical Sciences Jinan People's Republic of China

## Abstract

Chest CT images of the patient in 2016, 2017, 2018, and 2019. The lesion was significantly larger compared with its size in 2016

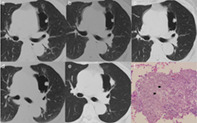

A 62‐year‐old woman was diagnosed with pulmonary bullae accompanied by pulmonary nodule at a local hospital. The patient presented with an intermittent cough at the outpatient department of the Cancer Hospital Affiliated with Shandong First Medical University in 2016. Routine blood examination revealed slightly elevated neutrophil counts, no hepatitis B or hepatic function abnormalities, and negativity for carcinoma embryonic antigen and squamous cell carcinoma‐associated antigens.

The expert multidisciplinary team (MDT) for pulmonary nodule at our hospital examined the computed tomography (CT) image from the other hospital (Figure 1a) and observed a nodule with smooth margin in the pulmonary bullae, consistent with a fungal ball formed by a fungal infection. The subsequent galactomannan (GM) test was positive, and the patient was diagnosed with fungal infection by the MDT. Oral antibiotics and regular re‐examinations were suggested to the patient. The patient's symptoms were significantly relieved. A re‐examination in 2017 (Figure 1b) revealed no significant changes in the intrapulmonary lesion from the initial diagnosis. The lesion slowly grew in 2018 (Figure 1c) and 2019 (Figure 1d). Observation of the pulmonary nodule was continued by the expert MDT at our hospital.

**FIGURE 1:**
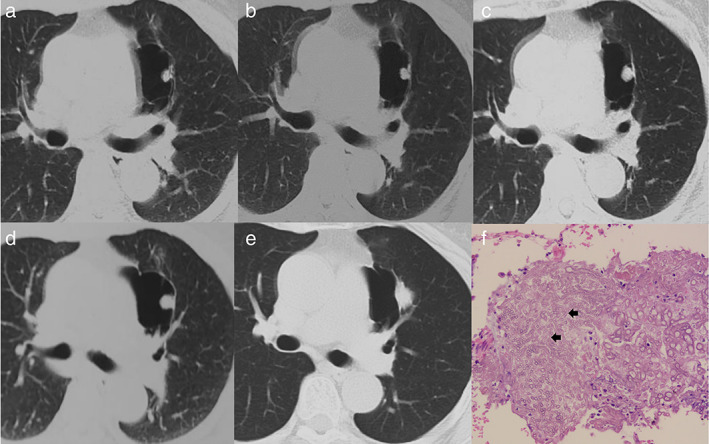
(a)–(d) Chest CT images of the patient in 2016, 2017, 2018, and 2019. (e) The lesion was significantly larger compared with its size in 2016. (f) Postoperative pathology indicated focal alveolar epithelial hyperplasia complicated by high levels of acute and chronic inflammatory cell infiltration, fibrosis, and hyphae (black arrow) in the lesion

In April 2020 (Figure 1e), the lesion was significantly larger compared with its size in 2016. Contrast‐enhanced CT revealed uneven enhancement of the lesion, with CT values of 27, 41, and 39 HU in the plain scan, arterial phase, and venous phase, respectively. Surprisingly, the nodule moved from the pulmonary bullae, its volume increased, and its margin became blurred. This abnormal behavior attracted the attention of the expert MDT for pulmonary nodules, and the patient was diagnosed with atypical lesion. Surgical resection was recommended. The patient underwent wedge resection of the upper left lobe in October 2020. Postoperative pathology indicated focal alveolar epithelial hyperplasia complicated by high levels of acute and chronic inflammatory cell infiltration, fibrosis, and hyphae in the lesion (Figure 1f). Combined with the clinical characteristics, the lesion was eventually diagnosed as an “*Aspergillus* ball”.

A fungal ball formed by *Aspergillus* infection is sometimes misdiagnosed as lung cancer. A nodule with a smooth margin in the pulmonary bullae is characteristic of a fungal ball formed by *Aspergillus* infection, and the lesion can enlarge slowly. At the initial diagnosis, this patient was correctly diagnosed by the expert MDT for pulmonary nodules at our hospital based on typical imaging manifestations, a positive GM test, and the relief of clinical symptoms after treatment with oral antibiotics.

In this case, the enlargement of the intrapulmonary nodule and “crawling out” of pulmonary bullae is rare imaging manifestations of fungal ball that have not been previously reported. This case report can provide a reference for the differential diagnosis of a fungal ball.

